# Street Connectivity is Negatively Associated with Physical Activity in Canadian Youth

**DOI:** 10.3390/ijerph8083333

**Published:** 2011-08-16

**Authors:** Graham Mecredy, William Pickett, Ian Janssen

**Affiliations:** 1 Department of Community Health and Epidemiology, Queen’s University, 99 University Avenue, Kingston, ON K7L 3N6, Canada; E-Mails: grahammecredy@gmail.com (G.M.); will.pickett@queensu.ca (W.P.); 2 Clinical Research Center, Angada 3, Kingston General Hospital, 76 Stuart St., Kingston, ON K7L 2V7, Canada; 3 School of Kinesiology and Health Studies, Queen’s University, 99 University Avenue, Kingston, ON K7L 3N6, Canada

**Keywords:** adolescent, physical activity, built environment, street connectivity

## Abstract

Street connectivity, defined as how well streets connect to one and other and the density of intersections, is positively associated with active transportation in adults. Our objective was to study the relation between street connectivity and physical activity in youth. Study participants consisted of 8,535 students in grades 6–10 from 180 schools across Canada who completed the 2006 Health Behaviour in School-aged Children (HBSC) survey. Street connectivity was measured in a 5 km circular buffer around these schools using established geographic information system measures. Physical activity performed outside of school hours was assessed by questionnaire, and multi-level regression analyses were used to estimate associations with street connectivity after controlling for several covariates. Compared to students living in the highest street connectivity quartile, those in the second (relative risk = 1.22, 95% confidence interval = 1.10–1.35), third (1.25, 1.13–1.37), and fourth (1.21, 1.09–1.34) quartiles were more likely to be physically active outside of school. In conclusion, youth in neighbourhoods with the most highly connected streets reported less physical activity outside of school than youth from neighbourhoods with less connected streets. Relationships between street connectivity and physical activity reported in this national study are in the opposite direction to those previously observed for active transportation in adult populations.

## 1. Introduction

A physically active lifestyle is an important determinant of the physical and mental health of children and youth [1]. Less than 10% of children and youth in Canada and the United States accumulate enough physical activity to meet current public health guidelines of 60 minutes per day of moderate-to-vigorous physical activity [2,3]. A lack of physical activity, therefore, represents a significant public health issue for the paediatric population.

To develop effective public health policies and interventions to improve the physical activity levels of our children and youth it is essential to understand the determinants of this behaviour. Research on the determinants of physical activity has, within recent years, begun to focus on the built environment [4,5]. The built environment is comprised of aspects of the physical surroundings in which we live our daily lives. These physical surroundings can help or hinder our desire and ability to engage in physical activity. One aspect of the built environment that may impact physical activity is a construct called street connectivity. Street connectivity refers to the directness of links and density of connections (*i.e.*, intersections) in street networks. A neighborhood with a highly connected street network has streets with many short links, numerous intersections, and few dead-ends and cul-de-sacs [6]. Highly connected street networks could make it more efficient and easier to walk or bicycle from one place to another (e.g., more direct routes, shorter travel distance).

Several studies have demonstrated that adults living in neighbourhoods with highly connected streets have higher active transportation levels by comparison to adults living in neighbourhoods with poorly connected streets [4,5,7,8]. Because active transportation, and walking in particular, is the most common physical activity that adults engage in [9,10], and because it would be difficult to engage in active transportation in neighbourhoods where street connections are limited, it makes sense conceptually that street connectivity is positively associated with physical activity in adult populations.

Children and youth participate in different types of physical activity than adults do. Active transportation for exercise is less common in young people, while unorganized sport and play are far more common [10]. To illustrate, playing hockey on the street (street hockey) is a common physical activity in Canadian children and youth, and is an example of how children and youth can use the streets in their neighbourhood to engage in physical activity outside of active transportation. Highly connected streets are more common in areas with a high population density, which corresponds with greater traffic density [11], which may in turn increase safety concerns around allowing children and youth to play outdoors. Therefore, from a theoretical standpoint, high street connectivity may discourage physical activity participation in children and youth, while cul-de-sacs and other features of poorly connected streets may provide a playground of sorts for children and youth to use for sport and play.

To our knowledge five studies have examined the relation between measures of street connectivity and the overall physical activity levels of young people [12–16]. The results of these studies are conflicting. In a sample of 98 adolescents (mean age = 16 years), Kligerman *et al.* [12] and Leung *et al.* [15] reported that a neighbourhood walkability index was positively associated with physical activity. Norman *et al.* [14] and Boone-Heinonen and Gordon-Larsen [16] found that street connectivity measures were negative correlates of moderate-to-vigorous activity within girls, but that no associations were present in boys. Finally, in a sample of 1,123 youth in grades 7–12, Mota *et al.* [13] found no association between street connectivity, as perceived by the study participants, and physical activity levels.

The objective of this study was to examine the relationship between an objective and comprehensive measures of street connectivity with physical activity in a national sample of Canadian youth. We accomplished this by linking individual records from a national survey to geographical information systems (GIS) measures of street connectivity that were obtained in the neighbourhoods of the survey participants. Our hope was that the findings from this national study would help inform the development of public health and urban planning interventions and policies aimed at improving the physical activity levels of children and youth.

## 2. Experimental Section

### 2.1. Overview of Study Sample and Design

Individual-level data on physical activity and potential covariates were gathered from the 2006 Canadian Health Behaviour in School-aged Children Survey (HBSC). The HBSC survey is a cross-national survey conducted in affiliation with the World Health Organization. The 2006 Canadian HBSC survey includes information on health behaviors, health outcomes, and contextual determinants of health among students in grades 6–10 (approximates ages of 11–15 years) from publicly funded schools sampled from all Canadian provinces and territories. A systematic single stage cluster sample, following guidelines in the international HBSC protocol, was used to identify the sampling unit of classes [17]. The sample excluded students enrolled in private schools, special needs schools, those who were home-schooled, as well as students that were absent on the day of the survey. Ethics approval was granted by the General Research Ethics Board of Queen’s University. Consent was provided by individual schools and their school boards, parents/guardians, and the student participants.

The original sample contained 9,672 students from 186 schools. Of these, 6 schools were excluded due to record linkage errors. An additional 664 students were missing data on either the physical activity outcome of interest or one of the covariates in the final model, and were subsequently deleted. This left 8,535 students (88.2%) from 180 schools (96.8%) available for analysis. There was no difference in the distribution of individual-level variables or prevalence of physical activity between excluded and included students (data not shown), implying that the 8,535 participants studied were representative of the original sample.

In addition to the individual-level data that were collected in the HBSS student survey, area-level data were collected on street connectivity and potential covariates around the schools of the HBSC participants using GIS. The area-level data was collected in the 5 km circular buffer surrounding each of the 180 schools. The 5 km buffer size has been applied successfully in previous Canadian HBSC studies [18–21] and is thought to be inclusive of the residential neighborhood of most students attending each school. Using school addresses and unique identifiers, individual-level data from the HBSC questionnaire were linked with the area-level data for analytical purposes.

### 2.2. Measurement of Street Connectivity

The CanMap Streetfiles (DMTI Spatial Inc., version 2008.3) GIS database was used to compile area-level measures of street connectivity using ArcGIS software (ESRI, version 9.3). This GIS database contains a precision built street map with accurate geospatial data on an array of geographical indicators. Each school was identified on the CanMap Streetfiles map using a combination of a preexisting school address layer and geocoding techniques. A network layer of intersection nodes was created and added to the 5 km buffer Streetfiles map of each school. Erroneous nodes were manually deleted before the street connectivity measures were obtained, as explained below.

**Figure 1.**
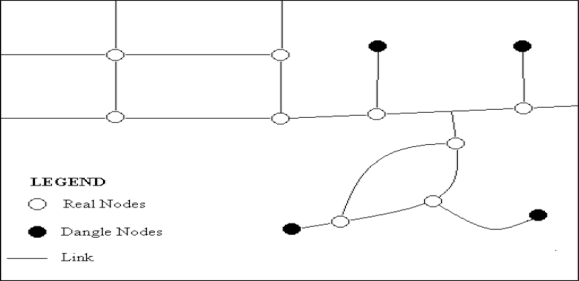
Components of the street connectivity measures. *Notes*: Real Node = the endpoint of a link that connects to other links (an intersection), Dangle Node = the endpoint of a link that has no other connections such as a dead-end or cul-de-sac, Link = a street segment between two nodes.

Three standard street connectivity measures were obtained: intersection density, average block length, and connected node ratio (Figure 1) [6]. Intersection density refers to the number of intersections per area [6], and is calculated by dividing the number of real nodes by the total land area. A higher number indicates more intersections and higher street connectivity. Average block length is the mean length of blocks in the area, and is calculated by dividing the sum of the link length per area by the number of nodes per area. Shorter blocks mean more intersections, and a greater number of routes available, showing higher street connectivity. Connected node ratio is the number of street intersections divided by the number of intersections plus cul-de-sacs [6], and is calculated by dividing the number of real nodes by the total number of nodes. The maximum value for this variable is 1, with higher numbers indicating that there are few cul-de-sacs and dead ends and higher connectivity.

**Figure 2.**
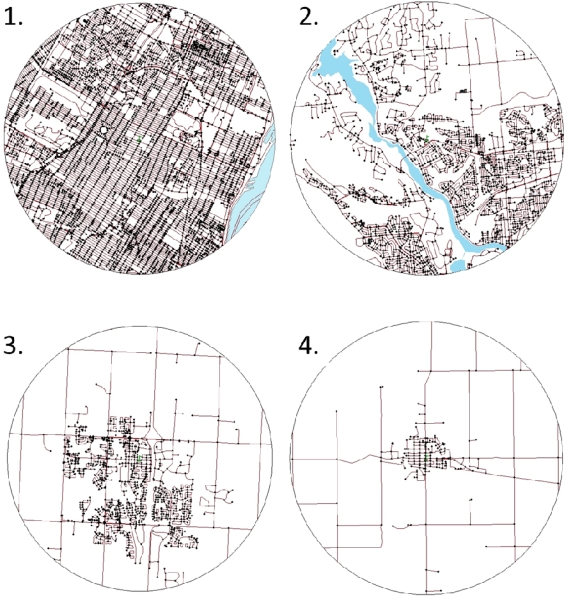
Example neighbourhoods from the different street connectivity quartiles. *Notes*: 1 = highest street connectivity quartile, 2 = second street connectivity quartile, 3 = third street connectivity quartile, and 4 = lowest street connectivity quartile.

We created a composite street connectivity scale based on the three individual street connectivity measures. Each of the 180 schools was ranked on each of the three measures. Principal component factor analysis revealed agreement between the three ranked variables; factor loadings were 0.96, 0.95, and 0.73 for the intersection density, average block length, and connected node ratio variables, respectively (Cronbach’s alpha = 0.86). These ranked variables were combined with equal weight to create the composite street connectivity measure, which was divided into quartiles. Participants in the first quartile resided in neighbourhoods with the highest street connectivity; participants in the fourth quartile lived in areas with the lowest street connectivity (Figure 2).

### 2.3. Measurement of Physical Activity

The study outcome was participation in moderate-to-vigorous intensity physical activity occurring outside of school. We chose not to assess overall physical activity as the outcome measure because a large proportion of a young person’s overall physical activity is accumulated at school [22], and because physical activity accumulated at school should not be affected by the connectivity of the surrounding streets. Conversely, physical activity accumulated outside of school, much of which would be in the youth’s home neighborhood, could conceptually be linked to street connectivity.

Responses to the HBSC question “*outside school hours: how many hours do you usually exercise in your free time so much that you get out of breath or sweat*?” were used to measure physical activity. Ordinal responses to this question were categorized to create a dichotomous outcome of ≥4 hours/week or <4 hours/week. The 4 hours/week threshold was based upon previous Canadian HBSC studies of the built environment and physical activity [19]. Total physical activity is accumulated in a variety of settings (*i.e.*, inside and outside of school), and accumulating 4 hours/week of physical activity outside of school is consistent with public health guidelines of 60 minutes/day of total moderate-to-vigorous activity as the remaining 3 hours of physical activity would be expected to occur at school [19].

### 2.4. Measurement of Potential Covariates

Variables considered *a priori* as potential covariates at the individual-level were: gender, grade, family socioeconomic status (SES), perceived neighborhood safety, and perceived neighborhood aesthetics. Potential area-level covariates were neighborhood-level SES, urban/rural geographic location, and parks and recreational facilities.

Gender and grade were self-reported. Family SES was gathered using the validated family affluence scale, which is comprised of four equally weighted items: family vehicle ownership, having a bedroom for yourself, family vacations during past year, and computer ownership (Cronbach’s alpha = 0.39) [23]. To measure perceived neighborhood safety, student responses to three questions were used: “*I feel safe in the area where I live*” (always, most of the time, sometimes, rarely or never*),* “*do you think that the area in which you live is a good place to live?*” (it’s really good, it’s good, it’s ok, it’s not very good/it’s not good at all)*,* and “*it is safe for younger children to play outside during the day?*” (strongly agree, agree, neither agree nor disagree, disagree, strongly disagree). These items were combined into a score with equal weight, and subsequently divided into quintiles (Cronbach’s alpha = 0.69) [19]. Neighborhood aesthetics were collected via answers to questions regarding how much litter, broken glass and garbage was present in their neighborhood (*none, some, lots*) and to what extent there were run-down houses and buildings in their neighborhood (*none, some, lots*).

Area-level SES was estimated using data from the 2001 Canadian Census using PCensus software [24] by combining ranked scores for median household income, employment rate and the proportion of the population with greater than high school education (Cronbach’s alpha = 0.70) [24]; schools were subsequently divided into quintiles. Geographic location was obtained from Statistics Canada data [24]. Schools were divided into urban schools in metropolitan areas, urban schools outside metropolitan areas, and rural schools [19]. The number of parks, trails, and recreational facilities were counted within the 5 km buffer around each school using CanMap Streetfiles [19]. A composite scale that considered the overall neighborhood recreational environment was constructed by combining ranked scores for each of the parks/facilities [19]; schools were subsequently divided into quintiles.

### 2.5. Statistical Analysis

Statistical analyses were performed in SAS version 9.2 (SAS Inc., Carry, NC). Distributions of key variables were characterized using conventional descriptive statistics. Bivariate multilevel models were fit to describe the relationship between measures of street connectivity and physical activity. We then developed a hierarchical series of multivariate models following a systematic approach: (1) Model 1 controlled for all individual-level (level 1) covariates; (2) Model 2 controlled for all individual-level and area-level (level 2) covariates; (3) Model 3 was fit using individual-level and area-level covariates; non-significant (p < 0.05) covariates were removed from the model using backwards elimination selection. Model 3 therefore considered the street connectivity scale, as well as a parsimonious list of covariates that significantly contributed in the final model (gender, grade, family SES, perceived safety, and perceived neighborhood condition). P-values for trends for all categorical variables were estimated by treating ordinal variables as continuous in the models.

The SAS GLIMMIX procedure was used to fit generalized linear models with a binomial distribution and a logit link, in order to account for the clustered (by school) and hierarchical nature of the data. These models used a Newton-Raphson with ridging technique to aid convergence. Cross-level interactions between street connectivity and gender, grade, and urban location were suspected, however, upon conducting likelihood ratio tests for interaction, none were identified.

Since the outcome of physical activity is common (>10%), odds ratios (OR) obtained from the regression models were converted to relative risks (RR) using the following equation [25], RR = OR/[(1 − P) + (OR × P)], where P is the prevalence of physical activity in the unexposed or referent group.

Population attributable risk (PAR) was calculated to indicate the proportion of reported physical activity that was attributed to living in an area with lower street connectivity. The PAR was calculated based on the results of Model 3 using the following equation, PAR = Pe(RR − 1)/(1 + Pe(RR − 1)), where Pe is the prevalence of exposure in the population [26]. PAR was calculated for each of the three lower street connectivity quartiles and then summed to obtain an overall estimate.

Finally, a sensitivity analysis was conducted within a subset of grade 9–10 students who reported additional variables that described neighbourhood characteristics. We determined whether each of self-reported vehicle traffic, stoplights/stop signs, and bike lanes/sidewalks in school neighbourhoods mediated the relationship between street connectivity and physical activity outside of school hours.

## 3. Results and Discussion

### 3.1. Results

Distributions of participants according to individual-level and area-level characteristics are shown by level of street connectivity in Table 1. There were noticeable and statistically significant differences in street connectivity according to family SES, neighborhood safety, amounts of litter in neighborhoods, and rundown homes in neighborhoods. For example, 40.7% of students in the lowest family SES group resided in a neighborhood in the highest street connectivity quartile as compared to 23.8% of students in the highest family affluence group (p < 0.0001). Level 2 (area-level) measures of SES were also associated with street connectivity such that higher SES areas had higher street connectivity scores. Geographic location was associated (p < 0.0001) with street connectivity; all students in the highest connectivity quartile resided in an urban core.

A description of physical activity levels in the total sample and by gender and grade is presented in Table 2. Of the total sample, 5.5% participated in no physical activity outside of school hours, 37.0% participated in at least 4 hours per week of physical activity outside of school, and 16.4% participated in at least 7 hours per week of physical activity outside of school. A lower percentage of those who participated in no physical activity outside of school were males than females (44.6% *vs.* 55.4%), while a higher percentage of those who participated in at least 4 hours per week of physical activity outside (54.9% *vs.* 45.1%) and at least 7 hours per week of physical activity outside of school (59.4% *vs.* 40.6%) were males than females. Physical activity levels outside of school were slightly higher in Grade 6–8 than Grade 9–10 students. For instance, while 57.7% of those who accumulated less than 4 hours per week of physical activity outside of school were comprised of Grade 6–8 students, only 54.8% of those who accumulated 4 or more hours per week of physical activity outside of school were comprised of Grade 6–8 students.

Of the total sample, 26.9% (n = 2,296) were in street connectivity group 1 (highest connectivity), 21.7% (n = 1,851) were in street connectivity group 2, 27.8% (n = 2,374) were in street connectivity group 3, and 23.6% (n = 2,014) were in street connectivity group 4 (lowest connectivity). The percentage of the sample that were physically activity (*i.e.*, 4 hours per week of physical activity outside of school) within each of the street connectivity groups ranged from a low of 30.7% in street connectivity group 1 to a high of 38.6% in street connectivity group 4 (Table 3). Table 3 lists the bivariate relations between the three street connectivity measures and the overall street connectivity scales with physical activity. All three connectivity measures, as well as the composite street connectivity scale, were associated with physical activity in a consistent fashion. For the overall street connectivity scale, by comparison to group 1 (highest connectivity), the relative risks of being physically active outside of school hours were higher in group 2 (RR: 1.29, 95% CI: 1.16–1.42), group 3 (RR: 1.31, 95% CI: 1.18–1.44), and group 4 (RR: 1.26, 95% CI: 1.14–1.40). The three components of the street connectivity scale (connected node ratio, intersection density, and average block length) were also related to the physical activity outcome such that the relative risks were significantly increased in groups 2, 3 and 4 by comparison to group 1. Note that the relations presented in Table 3 are bivariate relations. In other words, the relative risks presented in this table were not adjusted for any of the confounding variables.

Table 4 presents the associations between the covariates and the physical activity outcome. Of the individual-level (Level 1) covariates, gender, grade, family SES, perceived neighborhood safety, and perceived litter in the neighborhood were all significant independent predictors of physical activity outside of school hours (see Multivariate Model 3). While the geographic location and parks/recreational facilities area-level (Level 2) covariates were related to physical activity in the bivariate models, they were no longer significant in multivariate model 2, implying that they were not independent predictors of physical activity. Similarly, the perceived rundown homes and area-level SES variables were not related to physical activity.

The results of the multivariate model building process for the association between street connectivity and physical activity is also shown in Table 4. Street connectivity was significantly associated with physical activity outside of school hours. This relationship was consistent between the bivariate and three multivariate models. In other words, street connectivity remained a significant predictor of physical activity outside of school hours after adjustment for salient covariates (gender, grade, family SES, perceived safety, and perceived litter). The final multivariate model (model 3) suggested that, compared to students living in the first (highest) street connectivity quartile, those in the second (RR: 1.22; 95% CI: 1.10–1.35), third (RR: 1.25; 95% CI: 1.13–1.37), and fourth (lowest; RR: 1.21; 95% CI: 1.09–1.34) street connectivity quartiles were significantly more likely to be physical activity for 4 hours per week outside of school hours.

Based on the RR estimates provided for the street connectivity scale in multivariate model 3 in Table 4, and the prevalence of the study sample in the different street connectivity groups (Table 3), the population attributable risk for the physical activity outcome was calculated as following: [21.7%(1.22 − 1)/(1 + 21.7%(1.22 − 1)] + [27.8%(1.25 − 1)/(1 + 27.8%(1.25 − 1)] + [23.6%(1.21 − 1)/(1 + 23.6%(1.21 − 1)]. This population attributable risk calculation suggested that 15.8% (95% CI: 7.7–23.8) of the physical activity outcome in the study sample was attributable to not living in the most highly connected street connectivity group (group 1). In other words, had none (0%) of the sample been in the most highly connected street connectivity group (group 1), the prevalence of physical activity in the sample would have been 15.8% higher.

Table 5 presents a sensitivity analysis conducted within a subset of the HBSC survey. This subset consisted of 2,922 English speaking grade 9 and 10 students from the province of Ontario in whom supplemental information on vehicle traffic, stoplights/stop signs, and bike lanes/sidewalks was captured. As shown in the final multivariate model (model 2), high levels of vehicle traffic (RR: 0.87, 95% CI: 0.76–0.98) and the presence of stoplights or stop signs at busy intersections (RR: 1.16, 95% CI: 1.01–1.30) were related to the physical activity outcome, albeit in opposite directions. Conversely, the availability of bicycle lanes and sidewalks was not related to the physical activity outcome (p = 0.78 from bivariate model). A comparison of the RR estimates for the street connectivity scale in multivariate model 1 and multivariate model 2 indicate that that adjustment for the vehicle traffic and stoplights/stop sign measures did not alter the associations between the street connectivity and physical activity measures.

### 3.2. Discussion

Youth from neighbourhoods with lower street connectivity scores (*i.e.*, quartiles 2–4) were more likely to be physically active outside of school than youth from neighbourhoods with the highest street connectivity scores (*i.e.*, quartile 1). There appeared to be a threshold effect for street connectivity as the relative risks for physical activity, while different in the highest street connectivity quartile, were quite similar in each of the lower three quartiles. The population attributable risk estimates suggest that 15.8% of the physical activity outcome in the study sample was explained by street connectivity. Thus, from a public health perspective street connectivity has a meaningful impact on the physical activity of young people.

In addition to street connectivity, several of the covariates that were examined in this study were independently associated with physical activity. Participants reporting a high perceived safety of their neighbourhood were 1.47 (95% CI: 1.34–1.59) times more likely to be physically active outside of school hours, participants from a high family SES were 1.45 (95% CI: 1.30–1.61) time more likely to be physically active outside of school, and girls were 0.73 (95% CI: 0.86–0.77) times less likely to be physically active outside of school. Our final models controlled for the aforementioned factors.

The threshold effect we observed for the street connectivity exposure on the physical activity outcome is an important finding. Youth in the high street connectivity quartile were less likely to be physically active, and closer examination revealed that each of the schools in this quartile was located in the urban core of a large Census Metropolitan Area (e.g., Toronto, Montreal, Vancouver). While schools in the second most connected quartile were mainly from urban cores as well, these schools were located in less densely populated urban cores. Therefore, students living in the most highly populated urban cores reported considerably lower levels of physical activity outside of school hours than their peers. Based on the street connectivity illustrations shown in Figure 2, it is clear that the 5 km buffers around the schools capture vastly heterogeneous environments, with relatively dense street networks around most schools and varying levels of reductions in the street network density at further distances from the schools. By using the street connectivity score within the 5 km buffer as a proxy for the residential neighbourhood of all students attending that school, we do appreciate the fact that misclassification of our key study exposure occurred. Thus, the associations between street connectivity and physical activity that were observed in our study were likely underestimated.

Other factors, such as vehicle traffic, may have influenced the lower levels of physical activity reported by students living in the most highly connected neighbourhoods. Increased traffic in highly populated and connected neighbourhoods could lead to parent and youth concerns of outdoor safety and subsequently to a decrease in youth physical activity participation. While perceived vehicle traffic was related to physical activity levels in the subset of the study sample in whom these vehicle traffic measures were obtained (Table 5), adjustment for vehicle traffic in the multivariate model did not alter the affect estimates for street connectivity. Therefore, perceived vehicle traffic did not mediate or account for the relationship between street connectivity and physical activity. Furthermore, the perceived availability of bike lanes and sidewalks was not related to physical activity and did not mediate the relations between street connectivity and physical activity. This suggests that the relations between street connectivity and physical activity were not related to the active transportation component of physical activity.

Another potential explanation of the observed disparity is the lack of outdoor play space in neighbourhoods with highly connected streets. Homes in neighbourhoods with the highest connectivity are packed very tightly together (Figure 2), leaving little room for yards and driveways for young people to use for physical activity, which may lead to a decrease in outdoor activity. Also, the short blocks and lack of cul-de-sacs may make it difficult to play on the street. Poorly connected neighbourhoods with many cul-de-sacs present a space for youth to play, in a relatively safe and low traffic environment [11]. Future studies should consider the concept of outdoor space as a determinant of physical activity for youth residing in highly connected neighbourhoods, and attempt to characterize areas in which young people most often play outdoors.

Findings of this study are disparate from some of the five previous studies that examined the relationship between street connectivity and moderate-to-vigorous physical activity in youth. Mota *et al.* [13] reported no association, Norman *et al.* [14] and Boone-Heinonen and Gordon-Larsen [16] reported higher levels of physical activity in less connected areas for girls but not boys, and Kligerman *et al.* [12] and Leung *et al.* [15] reported higher levels of physical activity in more connected areas for both genders. The lack of consensus in these studies may be explained by their use of varying measures of connectivity, their study of highly specific geographic areas, and their comparatively small sample sizes. The size (n = 8,535), heterogeneity, nationally-representative nature, and use of a comprehensive and objective measures of street connectivity in our study is a methodological improvement on past research, and may explain why this study identified a different relationship.

The presence of lower levels of physical activity in highly connected neighborhoods is important as public health interventions that target youth physical activity levels have the potential to greatly impact population health. A challenge public health officials and urban planners will face when developing strategies for optimizing street connectivity is that the relations between street connectivity and physical activity for youth, as reported here, are in opposite direction to those previously reported for active transportation in adults [4,5,7,8]. Thus, the current public health and urbanist movement to create highly connected neighbourhoods [27], with the goal of increasing active transportation, may have a negative effect on the physical activity patterns of our youth. In light of these observations, consideration should be given to neighbourhoods that have a low street connectivity, but contain networks of pedestrian paths to increase their overall connectivity [28]. Development of neighbourhoods that are conducive to physical activity in all age groups, while challenging, have the potential to substantially ameliorate the health of the population.

Limitations of this study merit consideration. First, the cross-sectional nature of the HBSC data makes this research limited in its ability to determine the temporality of any observed relationship. With that being said, it is unlikely that active youth would be able to influence their family to move to less connected neighbourhoods to promote their physical activity, supporting the implied temporal sequence. Second, there is potential for area-level associations to be residually confounded by variables not captured in this research. An example of this is parental influences. Street connectivity influences adult physical activity [4,5,7,8], and parental physical activity is known to be a determinant of childhood physical activity [29]. Ethnicity has also been shown to affect youth physical activity levels [29], and not accounting for this variable in the analyses may also have resulted in residual confounding. Third, the 95% confidence intervals surrounding the relative risk estimates, as well as population attributable risk estimates derived from these relative risk values, may be biased slightly relative to more directly measured estimates of relative risk and associated population attributable risk estimates. Fourth, there is the possibility of misclassification on the basis of the street connectivity exposure. Since this research used a 5 km radius around the schools as a proxy for home neighbourhoods, this may have resulted in area-level characteristics being ascribed to students who in fact do not live within this radius. As no standard method exists for the measurement of neighbourhood environments, it was unclear what type of buffer should be used (radial *versus* street network buffers) and what radial distance around schools would be appropriate as a proxy to capture the participants’ home neighbourhood environment. It would be ideal to use a smaller buffer around participants’ homes; however, the Health Behaviour in School-Aged Children survey did not collect the necessary personal information on student addresses as it was an anonymous survey.

## 4. Conclusions

Physical activity patterns in 11–15 year old youth from the 2006 Health Behaviour in School-Aged Children survey were examined in association with neighborhood street connectivity measures, as measured using geographic information systems. Youth who resided in the neighborhoods in the highest street connectivity quartile were less likely to be physical activity outside of school than students living in neighborhoods in the remaining street connectivity quartiles. The relationship between street connectivity and physical activity in young people reported by this study is not consistent with relationships previously established for active transportation in adult populations. Society must be intentional in developing neighbourhoods that promote physical activity in all age groups.

## Figures and Tables

**Table 1.** Descriptive characteristics of the student sample and neighborhood environment (n = 8,535).

	**Street Connectivity Scale Quartiles**				

	**1 (highest)**	**2**	**3**	**4 (lowest)**	**p value**
***Level 1 variables***					
**Gender, %**					0.72
Male	25.8	23.1	27.7	23.4	
Female	27.8	20.5	27.9	23.8	
**Grade, %**					0.04
6	21.2	28.5	22.8	27.6	
7	33.3	14.5	27.6	24.6	
8	30.3	15.1	29.5	25.1	
9	23.2	27.4	30.3	19.2	
10	26.8	22.6	27.8	22.8	
**Family socioeconomic status, %**					<0.0001
low	40.7	17.4	20.7	21.2	
medium	27.8	19.3	27.2	25.7	
high	23.8	24.3	29.5	22.4	
**Perceived neighborhood safety, %**					<0.0001
low	32.7	20.0	26.8	20.5	
medium	26.4	22.0	28.8	22.8	
high	22.5	22.6	25.9	29.1	
**Perceived litter in neighborhood, %**					<0.0001
none	28.0	23.4	27.3	21.3	
some	25.8	20.2	28.3	25.7	
lots	25.3	16.9	29.0	28.8	
**Perceived rundown houses, %**					<0.0001
none	28.4	23.3	27.1	21.2	
some	21.2	16.0	30.6	32.2	
lots	26.7	14.3	28.1	31.0	

***Level 2 variables***					
**Socioeconomic status, %**					<0.0001
1 (lowest)	4.8	9.3	28.6	57.3	
2	39.3	13.2	28.4	19.1	
3	28.2	21.1	30.9	19.9	
4	35.8	33.7	14.6	15.9	
5 (highest)	22.7	30.5	33.9	9.1	
**Geographic location, %**					<0.0001
Urban, inside census metro area	43.8	32.6	20.7	2.9	
Urban, outside census metro area	0	7.0	43.2	49.8	
Rural	0	0	33.1	66.9	
**Number of parks and recreational facilities, %**					<0.0001
1 (least)	2.4	5.8	30.2	61.6	
2	3.9	7.4	50.9	37.9	
3	11.9	16.7	53.8	17.5	
4	47.1	43.2	5.9	3.8	
5 (most)	66.4	33.6	0	0	

*Note*: Row percents are reported for each of the variables listed in the table.

**Table 2.** Description of physical activity by gender and grade (n = 8,535).

	**Total**	**Male**	**Female**	**Grade 6–8**	**Grade 9–10**
**Physical activity outside of school hours, %**					
None	5.5	44.6	55.4	50.2	49.8
0.5 hours/week	12.5	36.8	63.2	60.4	39.6
1 hour/week	18.4	40.1	59.9	60.4	39.6
2–3 hours/week	26.7	44.4	55.6	56.2	43.9
4–6 hours/week	20.5	51.3	48.7	54.6	45.4
7+ hours/week	16.4	59.4	40.6	55.2	44.8
**Physically active ≥4 hours/week outside of school hours, %**					
No	63.0	41.7	58.3	57.7	42.3
Yes	37.0	54.9	45.1	54.8	45.2

*Note*: Row percents are reported for each of the variables listed in the table.

**Table 3.** Bivariate relations between street connectivity and physically activity (n = 8,535).

	**n**	**% physically active**	**RR (95% CI)**
**Street connectivity scale**			
1 (highest connectivity)	2,296	30.7	1.00
2	1,851	39.8	1.29 (1.16–1.42)
3	2,374	39.5	1.31 (1.18–1.44)
4 (lowest connectivity)	2,014	38.6	1.26 (1.14–1.40)
P trend			<0.0001

**Connected node ratio**			
1 (highest connectivity)	2,114	31.7	1.00
2	2,104	35.8	1.12 (1.00–1.25)
3	2,174	39.8	1.25 (1.12–1.38)
4 (lowest connectivity)	2,143	40.4	1.27 (1.15–1.41)
P trend			<0.0001
**Intersection density**			
1 (highest connectivity)	2,142	32.9	1.00
2	2,123	37.2	1.14 (1.01–1.27)
3	2,151	40.8	1.28 (1.15–1.41)
4 (lowest connectivity)	2,119	37.0	1.14 (1.01–1.27)
P trend			0.008
**Average block length**			
1 (highest connectivity)	2,161	32.1	1.00
2	2,095	37.6	1.18 (1.05–1.31)
3	2,122	40.6	1.31 (1.18–1.45)
4 (lowest connectivity)	2,154	37.7	1.19 (1.06–1.33)
P trend			0.001

*Note*: RR (95% CI) = relative risk (95% confidence interval).

**Table 4.** Multivariate relations between street connectivity and physical activity (n = 8,535).

	**% Physically active**	**Bivariate Model RR (95% CI)**	**Multivariate Model 1 RR (95% CI)**	**Multivariate Model 2 RR (95% CI)**	**Multivariate Model 3 RR (95% CI)**
**Street connectivity scale**					
1 (high)	30.7	1.00	1.00	1.00	1.00
2	39.8	1.29 (1.16–1.42)	1.22 (1.09–1.35)	1.25 (1.12–1.39)	1.22 (1.10–1.35)
3	39.5	1.31 (1.18–1.44)	1.24 (1.12–1.37)	1.34 (1.15–1.53)	1.25 (1.13–1.37)
4 (low)	38.6	1.26 (1.14–1.40)	1.20 (1.08–1.33)	1.29 (1.09–1.51)	1.21 (1.09–1.34)
P trend		<0.0001	0.0009	0.002	0.0004

***Level 1 covariates***					
**Gender**					
Male	43.6	1.00	1.00	1.00	1.00
Female	31.2	0.71 (0.67–0.76)	0.73 (0.68–0.77)	0.72 (0.68–0.77)	0.73 (0.68–0.77)
P value		<0.0001	<0.0001	<0.0001	<0.0001
**Grade**					
6	35.2	1.00	1.00	1.00	1.00
7	34.7	1.01 (0.91–1.12)	1.01 (0.91–1.12)	1.01 (0.91–1.11)	1.02 (0.91–1.12)
8	37.2	1.07 (0.97–1.17)	1.10 (0.99–1.20)	1.08 (0.98–1.19)	1.10 (0.99–1.20)
9	37.9	1.08 (0.97–1.19)	1.11 (1.01–1.22)	1.10 (0.99–1.21)	1.11 (1.01–1.22)
10	39.2	1.12 (1.01–1.23)	1.16 (1.05–1.27)	1.14 (1.03–1.25)	1.16 (1.05–1.27)
P trend		0.02	0.001	0.003	0.0008
**Family socioeconomic status**					
low	26.4	1.00	1.00	1.00	1.00
medium	33.0	1.21 (1.07–1.36)	1.17 (1.03–1.32)	1.17 (1.03–1.32)	1.16 (1.03–1.31)
high	41.9	1.53 (1.37–1.69)	1.46 (1.30–1.62)	1.46 (1.31–1.63)	1.45 (1.30–1.61)
P trend		<0.0001	<0.0001	<0.0001	<0.0001
**Perceived neighborhood safety**					
low	29.7	1.00	1.00	1.00	1.00
medium	36.7	1.21 (1.12–1.31)	1.20 (1.10–1.30)	1.20 (1.11–1.30)	1.19 (1.10–1.29)
high	45.0	1.49 (1.37–1.61)	1.48 (1.35–1.61)	1.48 (1.36–1.61)	1.47 (1.34–1.59)
P trend		<0.0001	<0.0001	<0.0001	<0.0001
**Perceived litter in neighborhood**					
none	36.6	1.00	1.00	1.00	1.00
some	37.7	1.04 (0.98–1.10)	1.10 (1.03–1.16)	1.10 (1.03–1.17)	1.12 (1.05–1.18)
lots	35.7	1.01 (0.89–1.12)	1.10 (1.97–1.24)	1.10 (0.96–1.23)	1.13 (1.01–1.26)
P trend		0.38	0.007	0.007	0.0004
**Perceived rundown houses**					
none	36.7	1.00	1.00	1.00	
some	37.7	1.04 (0.96–1.11)	1.06 (0.98–1.14)	1.06 (0.98–1.15)	
lots	38.1	1.06 (0.88–1.25)	1.12 (0.92–1.33)	1.12 (0.92–1.34)	
P trend		0.27	0.07	0.07	

***Level 2 covariates***					
**Area socioeconomic status**					
1 (lowest)	38.5	1.00		1.00	
2	33.8	0.85 (0.74–0.97)		0.91 (0.80–1.03)	
3	37.7	0.96 (0.85–1.08)		1.03 (0.92–1.15)	
4	33.2	0.87 (0.76–0.99)		0.92 (0.81–1.05)	
5 (highest)	40.6	1.02 (0.91–1.15)		1.02 (0.90–1.15)	
P trend		0.60		0.57	
**Geographic location**					
Urban inside CMA	35.7	1.00		1.00	
Urban outside CMA	37.2	1.06 (0.96–1.17)		0.97 (0.84–1.10)	
Rural	40.7	1.14 (1.02–1.26)		1.01 (0.87–1.16)	
P trend		0.02		0.87	
**Parks/recreational facilities**					
1 (least)	39.0	1.00		1.00	
2	38.2	0.97 (0.85–1.10)		0.99 (0.87–1.11)	
3	37.1	0.91 (0.79–1.03)		0.94 (0.82–1.06)	
4	34.9	0.89 (0.78–1.02)		1.02 (0.88–1.17)	
5 (most)	35.7	0.88 (0.77–1.00)		1.13 (0.97–1.30)	
P trend		0.03		0.23	

*Notes*: RR (95% CI) = relative risk (95% confidence interval). Model 1 was adjusted for all individual-level covariates. Model 2 was adjusted for all individual-level and area-level covariates. Model 3 was adjusted for covariates that were p<0.05 in the model.

**Table 5.** Sensitivity analysis of the relations between street connectivity and physical activity in grade 9 and 10 students from Ontario (n = 2,922).

	**n**	**% Physically active**	**Bivariate Model RR (95% CI)**	**Multivariate Model 1 RR (95% CI)**	**Multivariate Model 2 RR (95% CI)**
**Street connectivity scale**					
1 (highest)	731	30.7	1.00 (referent)	1.00 (referent)	1.00 (referent)
2	728	39.8	1.13 (0.91–1.37)	1.08 (0.88–1.30)	1.09 (0.89–1.30)
3	725	39.5	1.24 (1.02–1.47)	1.19 (0.98–1.41)	1.19 (0.99–1.40)
4 (lowest)	738	38.6	1.12 (0.91–1.34)	1.09 (0.89–1.30)	1.09 (0.90–1.30)
P trend			0.20	0.27	0.24

***Additional variables***					
**Vehicle traffic**					
low	1,111	42.0	1.00 (referent)		1.00 (referent)
medium	925	38.7	0.93 (0.83–1.04)		0.94 (0.84–1.05)
high	886	35.6	0.86 (0.76–0.97)		0.87 (0.76–0.98)
P trend			0.01		0.02
**Stoplights or stop signs at busy intersections**					
no	521	34.4	1.00 (referent)		1.00 (referent)
yes	2,401	40.0	1.15 (1.02–1.30)		1.16 (1.01–1.30)
P value			0.03		0.03
**Bike lanes and sidewalks**					
no	934	38.1	1.00 (referent)		
yes	1,988	39.4	1.01 (0.91–1.12)		
P value			0.78		

*Notes*: RR (95% CI) = relative risk (95% confidence interval). Model 1 adjusted for significant covariates from Table 4. Model 2 adjusted for covariates in Model 1 and additional variables that were p < 0.05 (vehicle traffic, stoplights, or stop signs).
